# Recurrent gastric solitary Peutz–Jeghers polyp after endoscopic treatment: A case report and literature review

**DOI:** 10.1097/MD.0000000000045276

**Published:** 2025-10-17

**Authors:** Siying Huang, Yingying Yan, Dunhuang Peng

**Affiliations:** a Department of Gastroenterology, The Second Affiliated Hospital of Fujian Medical University, Quanzhou, Fujian Province, P.R. China.

**Keywords:** endoscopic submucosal dissection, hamartomatous polyp, Peutz–Jegher syndrome, recurrent gastric solitary Peutz–Jeghers polyp

## Abstract

**Rationale:**

Solitary Peutz-Jeghers polyp (SPJP) is a rare hamartomatous polyp that histologically mimics polyps seen in Peutz-Jeghers syndrome but occurs in isolation, without the associated mucocutaneous pigmentation, intestinal polyposis, or family history. Gastric involvement is exceedingly uncommon, with only 16 cases documented worldwide. This report aims to contribute a novel case that highlights a previously unreported clinical course for this rare entity.

**Patient concerns:**

A 49-year-old Chinese female presented with a primary complaint of abdominal distension.

**Diagnoses:**

Recurrent gastric SPJP.

**Interventions:**

The patient underwent 2 separate endoscopic submucosal dissection procedures. The first was for the initial resection of the antral polyp, and the second was performed 1 year later to remove the recurrent polyp at the original site.

**Outcomes:**

Both endoscopic submucosal dissection procedures were completed successfully without any immediate or periprocedural complications. The patient recovered well following each intervention. The pathological diagnosis of a Peutz-Jeghers hamartomatous polyp was consistent after both resections.

**Lessons:**

This case represents the first documented instance of a recurrent gastric SPJP. It demonstrates that even solitary, sporadic Peutz-Jeghers polyps may have a potential for recurrence. Therefore, we recommend complete endoscopic or surgical resection of these polyps, followed by stringent endoscopic surveillance to facilitate the early detection and management of recurrence. This finding provides crucial insight for the management and follow-up strategy of this rare condition.

## 1. Introduction

Solitary Peutz–Jeghers polyp (SPJP) is a distinct type of hamartomatous polyp that histologically resembles those found in Peutz–Jeghers syndrome (PJS); however, SPJP lacks the associated mucocutaneous pigmentation and a relevant family history.^[1]^ Although SPJP most commonly occurs in the small intestine, gastric manifestations are exceptionally rare. To date, only 16 cases of gastric SPJP have been reported, highlighting its clinical rarity and significance.

This case report presents a recurrent gastric SPJP in a 49-year-old female patient and provides a review of the relevant literature. The uniqueness of this case lies in its being the first documented instance of recurrent gastric SPJP. By analyzing this rare case, we aimed to offer insights into future clinical management and emphasize the importance of close long-term follow-up for affected patients.

## 2. Case report

**Figure 1.**
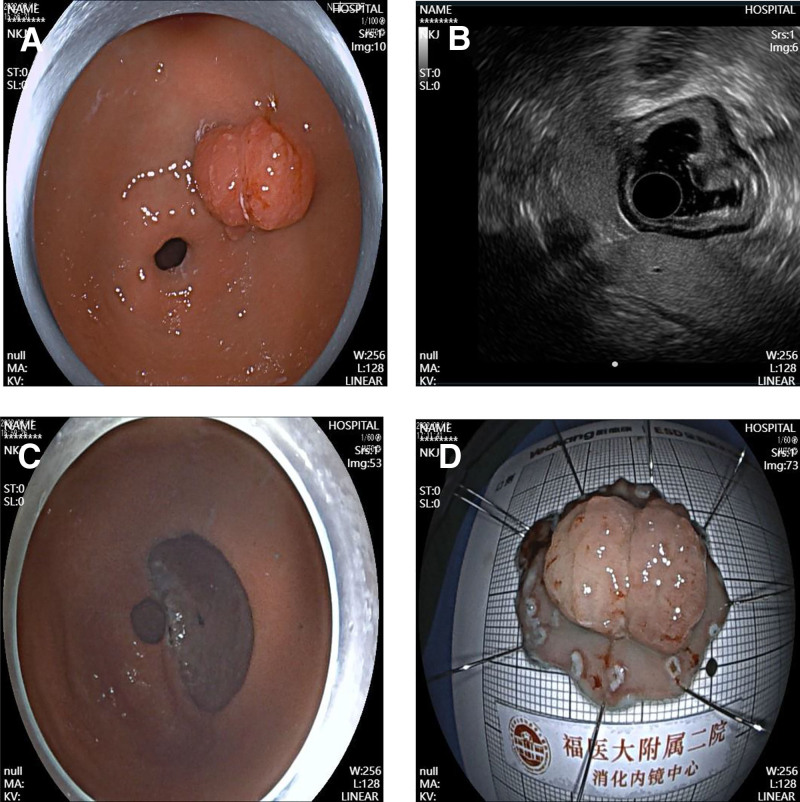
First endoscopic treatment. (A) Broad-based polypoid lesion on the posterior wall of the gastric antrum. (B) Endoscopic ultrasonography (EUS) showing a lesion originating from the mucosal layer with a heterogeneous hypoechoic pattern, partially fused with the muscularis mucosae while maintaining an intact submucosal layer. (C) Post-endoscopic submucosal dissection (ESD) wound. (D) Resected specimen after ESD.

**Figure 2.**
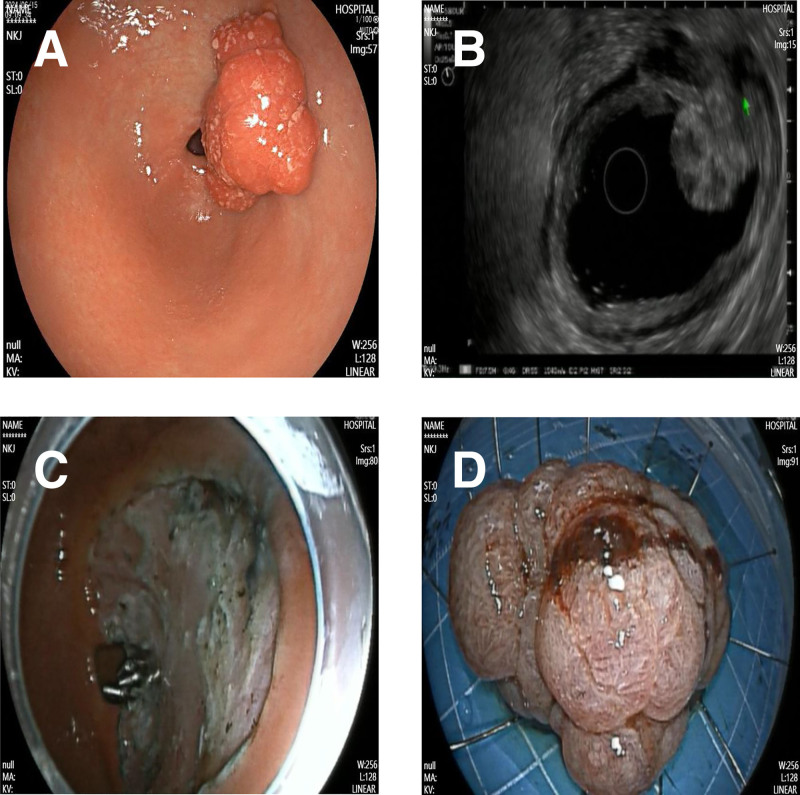
Second endoscopic treatment. (A) Recurrent broad-based polypoid lesion on the posterior wall of the gastric antrum. (B) Endoscopic ultrasonography (EUS) demonstrating a mucosal-originating lesion (2.2 × 2.1 cm in cross-section) with a heterogeneous hypoechoic pattern, showing fusion of the first 3 layers with indistinct boundaries and protrusion of the muscularis propria toward the mucosal surface. (C) Post-endoscopic submucosal dissection (ESD) wound. (D) Resected specimen after ESD.

**Figure 3.**
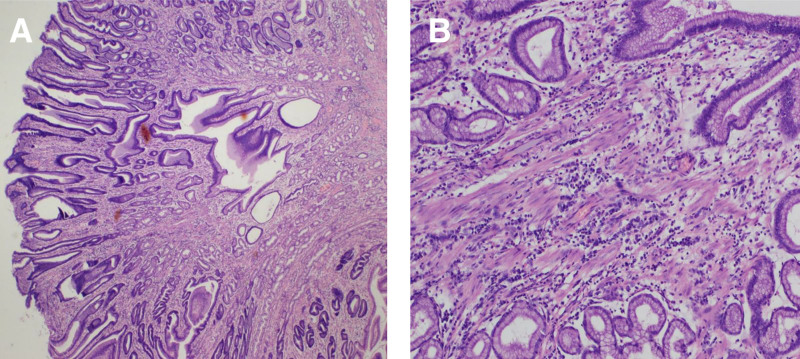
Hematoxylin and eosin (H&E) staining. (A and B) Histopathological examination showing characteristic arborizing proliferation of smooth muscle fibers extending centrally to the polyp apex. Each branching structure is covered with intact mucosa, creating a distinctive dendritic or villous architectural pattern under low-power magnification.

A 49-year-old female was admitted to the Department of Gastroenterology at the Second Affiliated Hospital of Fujian Medical University. The patient had no family history of polyps or tumors. Initial gastroscopy revealed a broad polypoid lesion on the posterior wall of the gastric antrum. Endoscopic ultrasonography further demonstrated a lesion measuring approximately 2 × 3 cm originating from the mucosal layer, with heterogeneous hypoechoic features. The lesion appeared partially fused with the muscularis mucosae and had ill-defined margins, while the submucosal layer remained intact. Based on these findings, the patient underwent endoscopic submucosal dissection (ESD), and the lesion was successfully resected (Fig. 1). One year later, during follow-up for recurrent epigastric pain, gastroscopy revealed a similar broad-based polypoid lesion at the same location in the gastric antrum. Endoscopic ultrasonography showed a lesion originating from the mucosa with a heterogeneous hypoechoic pattern, measuring approximately 2.2 × 2.1 cm in cross-section. Partial fusion of the first 3 layers was observed, with indistinct boundaries and protrusion of the muscularis propria toward the mucosal surface. Consequently, a second ESD was performed (Fig. 2). Both endoscopic resections were completed without complications. Histopathological examination of both specimens revealed hamartomatous polyps extending from the muscularis mucosae to the normally covered mucosal polyps, with characteristic arborizing bundles of smooth muscle fibers, features consistent with the diagnosis of a Peutz–Jeghers hamartomatous polyp (Fig. 3). The patient was discharged without complications and remained in good clinical condition throughout the 6-month follow-up period. The patient was satisfied with the diagnosis and treatment.

## 3. Discussion

Gastric SPJPs are exceptionally rare. Limited literature reports indicate that they predominantly occur in the small intestine, followed by the colorectal region.^[2]^ The first reported case of gastric SPJP was documented by Kuwano et al in 1989.^[3]^ To date, only 16 cases of gastric SPJP have been reported (Table 1). The clinical manifestations of SPJP are nonspecific and may include epigastric discomfort, gastrointestinal bleeding, and nausea.^[4]^ Reported cases show that gastric SPJPs vary in size, ranging from 5 mm to 150 mm.^[4,5]^ The age at diagnosis has been reported to range from 17^[3]^ to 81 years,^[4]^ with a median of 49 years. Most lesions have been identified in the gastric body and antrum, with only one reported case in the cardia.^[6]^ Notably, current case reports have demonstrated a higher incidence in female patients (Table 1). Diagnosis of SPJP is based on histopathological examination. The histological features are similar to those of Peutz–Jegher polyps and are characterized by arborizing smooth muscle proliferation within the core of the polyps. These muscle bundles extend toward the polyp apex, becoming progressively thinner near the surface, and are covered by mucosa along each branch. These features create a dendritic or villous architecture under low-power microscopy.^[6]^

**Table 1:** Cases of gastric solitary Peutz–Jeghers polyp.

Treatment	Authors	Year	Age	Gender	Size (mm)	Location
Endoscopic resection	Kuwano et al	1989	17	Man	20	Posterior wall of gastric body
	Grisendi et al	1990	53	Woman	20	Stomach
	O’Loughlin et al	2002	38	Woman	70 × 40	Stomach
	Oncel et al	2003	78	Man	5	Stomach
	Harbaum et al	2009	61	Man	10	Stomach
	Shi et al	2014	67	Man	25	Stomach
	Bai-Cang et al	2017	53	Man	110 × 80	Greater curvature of the gastric antrum
	Khsiba et al	2022	81	Man	40	Greater gastric curvature, gastric body
	Noelia Madera et al	2024	50	Woman	12	Gastric body
	Present case	2025	49	Woman	25 × 30/22 × 21	Antrum of stomach
Surgery	Hunt et al	1996	27	Woman	80	Distal Stomach
	Sakadamis et al	2001	47	Woman	75 × 50	Anterior wall of the antrum of the stomach
	Jin et al	2012	71	Woman	40 × 30	Antrum of stomach
	Lunca et al	2014	43	Woman	150 × 70X50	Cardias
	Yoshizawa et al	2016	37	Woman	65 × 60 × 30	Greater curvature of the upper gastric body
	Mongardini et al	2024	31	Woman	50 × 60	Gastric body, along the greater curvature
N/A	Goto et al	2020	32	Woman	10	Gastric body

PJS is an autosomal dominant disorder characterized by multiple hamartomatous polyps in various organs, including the stomach, small intestine, colon, and pancreas, as well as by distinctive mucocutaneous pigmentation, typically manifesting as perioral, labial, gingival, and acral melanotic macules.^[7]^ The pathogenesis of PJS is primarily associated with mutations in the STK11/LKB1 gene located on chromosome 19p13.3,^[8]^ which significantly increases the risk of cancer, including pancreatic, colorectal, gastric, breast, ovarian, testicular, and uterine.^[9–11]^ However, genetic analyses of 3 reported SPJP cases showed no STK11 mutations,^[2]^ suggesting that SPJP may represent a clinical entity distinct from PJS. Supporting the distinction, Oncel et al followed 8 SPJP patients (including one gastric case) for 11.5 years and observed no evidence of PJS-associated cancers.^[12]^ We recommended that the patient undergo STK11 genetic testing, but the patient declined. Although several duodenal SPJPs with malignant components have been reported,^[13–16]^ none of the 16 gastric SPJP cases has shown malignant features in resected specimens. Only one case was associated with a colonic high-grade tubular adenoma,^[17]^ and another with an intraductal papillary mucinous neoplasm, a known precancerous lesion of the pancreas.^[4]^ Due to the limited number of cases, the carcinogenic potential of gastric SPJP remains unclear.

Currently, no standardized treatment protocol exists for gastric SPJP. A review showed that 10 cases were treated with endoscopic resection, while 6 cases underwent surgical treatment. All patients reportedly achieved good outcomes without recurrence. Notably, our case represents the first reported recurrence after ESD. A pathological review of the initial resection confirmed negative margins, suggesting complete removal of the lesion. The mechanism underlying recurrence remains uncertain; however, it may involve genetic factors, warranting further investigation. The limitation of this case lies in the absence of relevant genetic testing. Given this uncertainty, close follow-up is essential to monitor potential recurrence and gather more clinical data.

## 4. Conclusions

Given the rarity of gastric SPJP, the potential cancer risk requires further research and clinical observation. Considering reports of malignant components in SPJPs at other sites, we recommend resection of gastric SPJPs as a precautionary measure. The choice between endoscopic and surgical approaches should consider the location and size of the lesion. Posttreatment endoscopic surveillance within 1 year is advisable to enable early detection of recurrence. In addition, long-term follow-up is crucial to deepen our understanding of this disease and to clarify the mechanisms underlying recurrence.

## Acknowledgments

We would like to thank Editage (www.editage.cn) for English language editing.

## Author contributions

**Resources:** Siying Huang.

**Writing – original draft:** Siying Huang, Yingying Yan, Dunhuang Peng.

**Writing – review & editing:** Siying Huang, Yingying Yan, Dunhuang Peng.

## References

[1] Liu BL Zhou H Risech M Ky A Houldsworth J Ward SC. Solitary peutz-jeghers type polyp of jejunum with gastric fundic and antral gland lining mucosa: a case report and review of literature. Int J Surg Pathol. 2022;30:539–42. 34955063 10.1177/10668969211067760

[2] Zou BC Wang FF Zhao G. A giant and extensive solitary Peutz–Jeghers-type polyp in the antrum of stomach: case report. Medicine (Baltim). 2017;96:e8466. 10.1097/MD.0000000000008466 PMC5728830 29245215

[3] Kuwano H Takano H Sugimachi K. Solitary Peutz–Jeghers type polyp of the stomach in the absence of familial polyposis coli in a teenage boy. Endoscopy. 1989;21:188–90. 2550212 10.1055/s-2007-1012939

[4] Khsiba A Bradai S Nakhli A. Solitary gastric Peutz–Jeghers polyp: a case report. Pan Afr Med J. 2022;41:65. 35371379 10.11604/pamj.2022.41.65.29526 PMC8933450

[5] Mongardini FM Nazzaro L Fuschillo G. Gentle giant? Giant gastric solitary Peutz–Jeghers polyp. Dig Dis Sci. 2024;69:349–54. 38183558 10.1007/s10620-023-08240-5

[6] Lunca S Porumb V Velenciuc N Ferariu D Dimofte G. Giant solitary gastric Peutz–Jeghers polyp mimicking a malignant gastric tumor: the largest described in literature. J Gastrointestin Liver Dis. 2014;23:321–4. 25267961 10.15403/jgld.2014.1121.233.vpb2

[7] Tacheci I Kopacova M Bures J. Peutz–Jeghers syndrome. Curr Opin Gastroenterol. 2021;37:245– 54. 33591027 10.1097/MOG.0000000000000718

[8] Westerman AM Wilson JH. Peutz–Jeghers syndrome: risks of a hereditary condition. Scand J Gastroenterol Suppl. 1999;230:64–70. 10499464 10.1080/003655299750025561

[9] Klimkowski S Ibrahim M Ibarra Rovira JJ. Peutz–Jeghers syndrome and the role of imaging: pathophysiology, diagnosis, and associated cancers. Cancers (Basel). 2021;13:5121. 34680270 10.3390/cancers13205121 PMC8533703

[10] Hearle N Schumacher V Menko FH. Frequency and spectrum of cancers in the Peutz–Jeghers syndrome. Clin Cancer Res. 2006;12:3209– 15. 16707622 10.1158/1078-0432.CCR-06-0083

[11] Giardiello FM Brensinger JD Tersmette AC. Very high risk of cancer in familial Peutz–Jeghers syndrome. Gastroenterology. 2000;119:1447– 53. 11113065 10.1053/gast.2000.20228

[12] Oncel M Remzi FH Church JM Goldblum JR Zutshi M Fazio VW. Course and follow-up of solitary Peutz–Jeghers polyps: a case series. Int J Colorectal Dis. 2003;18:33–5. 12458378 10.1007/s00384-002-0411-x

[13] Aneiros J Matamala M Garcia Del Moral R Lopez JJ Aguilar D Camara M. Hamartomatous solitary polyp with malignant progression in the jejunum. A histochemical and immunohistochemical study by light and electron microscopy. Acta Pathol Jpn. 1988;38:1031– 40. 3188911 10.1111/j.1440-1827.1988.tb02375.x

[14] Sekino Y Inamori M Hirai M. Solitary Peutz–Jeghers type hamartomatous polyps in the duodenum are not always associated with a low risk of cancer: two case reports. J Med Case Rep. 2011;5:240. 21707968 10.1186/1752-1947-5-240 PMC3141699

[15] Ichiyoshi Y Yao T Nagasaki S Sugimachi K. Solitary Peutz–Jeghers type polyp of the duodenum containing a focus of adenocarcinoma. Ital J Gastroenterol. 1996;28:95–7. 8782002

[16] Jamaludin AZ Telisinghe PU Yapp SK Chong VH. Solitary duodenal hamartomatous polyp with malignant transformation: report of a case. Surg Today. 2009;39:527–32. 19468811 10.1007/s00595-008-3873-9

[17] Harbaum L Geigl JB Volkholz H. Sporadic gastric Peutz–Jeghers polyp with intraepithelial neoplasia. APMIS. 2009;117:941–3. 20078560 10.1111/j.1600-0463.2009.02549.x

